# Pseudoprogression in the era of immunotherapy-based strategies for recurrent head and neck squamous cell carcinoma achieving complete response: A case report

**DOI:** 10.1097/MD.0000000000033797

**Published:** 2023-08-04

**Authors:** Jiawei Zhou, Jie Luo, Jiajia Gu, Jingyuan Zhang, Dayong Gu, Xiuming Zhang, Feijiang Wang, Xia He, Lirong Wu

**Affiliations:** a Department of Radiation Oncology, Jiangsu Cancer Hospital & Jiangsu Institute of Cancer Research & The Affiliated Cancer Hospital of Nanjing Medical University, Nanjing, China; b Department of Health Care, Jinling Hospital, Medical School of Nanjing University; c Department of Pathology, Jiangsu Cancer Hospital & Jiangsu Institute of Cancer Research & The Affiliated Cancer Hospital of Nanjing Medical University, Nanjing, China; d Department of Radiology, Jiangsu Cancer Hospital & Jiangsu Institute of Cancer Research & The Affiliated Cancer Hospital of Nanjing Medical University, Nanjing, China.

**Keywords:** case report, complete response, head and neck squamous cell carcinoma, immunotherapy, pseudoprogression

## Abstract

In the last few years, treatment of head and neck squamous cell carcinoma (HNSCC) has been enhanced by the emergence of immunotherapy. A biological phenomenon unique to immunotherapy is pseudoprogression, an increase in tumor burden or the appearance of a new lesion subsequently followed by tumor regression.

**Patient concerns:**

A 78-year-old man complaining of a lump (6*4 cm) gradually swelling on the right side of his neck with recurrent buccal mucosa squamous cell carcinoma presented to our institution. Two months prior, he received resection of the buccal lesion but refused suggested adjuvant chemoradiotherapy after the operation.

**Diagnoses:**

Recurrent buccal mucosa squamous cell carcinoma.

**Interventions:**

Induction immunotherapy was initiated, followed by a new node appearing on the surface of the neck mass. We considered the presence of pseudoprogression and continued with immunotherapy. The patient received immunotherapy combined with chemotherapy and intensity-modulated radiation therapy (IMRT) consecutively.

**Outcomes:**

The patient experienced an excellent recovery with the disappearance of pain and the lump, along with return of a healthy appetite, weight gain and positive outlook. Complete response (CR) was also noted by magnetic resonance imaging (MRI) scan, with the upper right neck mass significantly retreated to unclear display. The patient is still alive with stable, asymptomatic disease at the time of this writing.

**Lessons:**

These results provide confidence in the safety and efficacy of radical chemo-radio-immunotherapy for the treatment of recurrent, unresectable or metastatic HNSCC.

## 1. Introduction

As the sixth most common cancer in the world, over 500,000 new cases of head and neck squamous cell carcinoma (HNSCC) occur annually.^[1]^ Particularly for patients with recurrent/metastatic (R/M) HNSCC, the median survival is 6 to 12 months due to limited therapeutic options.^[2]^ In recent years, with the appearance of new strategies, immunotherapy has made a breakthrough in the treatment of HNSCC. Anti-programmed death 1 (PD-1) monoclonal antibodies combined with chemotherapy have demonstrated better overall survival in R/M HNSCC (13.0 months vs 10.7 months).^[3]^ Encouragingly, NCCN guidelines (version 3.2021) list pembrolizumab as the first-line preferred regimen for recurrent, unresectable or metastatic HNSCC. One rare but significant phenomenon associated with immunotherapy is pseudoprogression, which refers to an increase in tumor burden or the appearance of a new lesion followed by tumor regression.^[4]^ Here, we report a complete response (CR) case in a patient with recurrent HNSCC in which pseudoprogression was observed in immunotherapy-based treatments.

## 2. Case description

**Figure 1.**
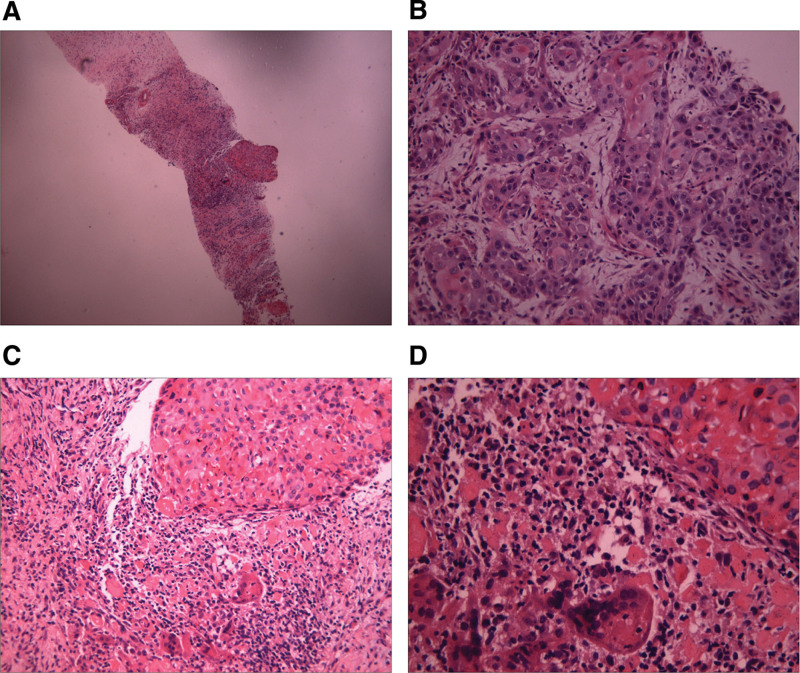
Postoperative pathology of the patient. A: Sample H&E × 40, revealing cancer nests and the landscape of immune microenvironment. B: Sample H&E × 200, revealing moderately differentiated keratinizing squamous cell carcinoma. C: Sample H&E × 200, revealing active immune microenvironment around squamous cell carcinoma nests. D: Sample H&E × 400, revealing TILs infiltration in cancer nests and different components of the immune microenvironment: monocytes, multinucleated giant cells, T-lymphocytes, B-lymphocytes. H&E = hematoxylin-eosin staining, TILs = tumor infiltrating lymphocytes.

**Figure 2.**
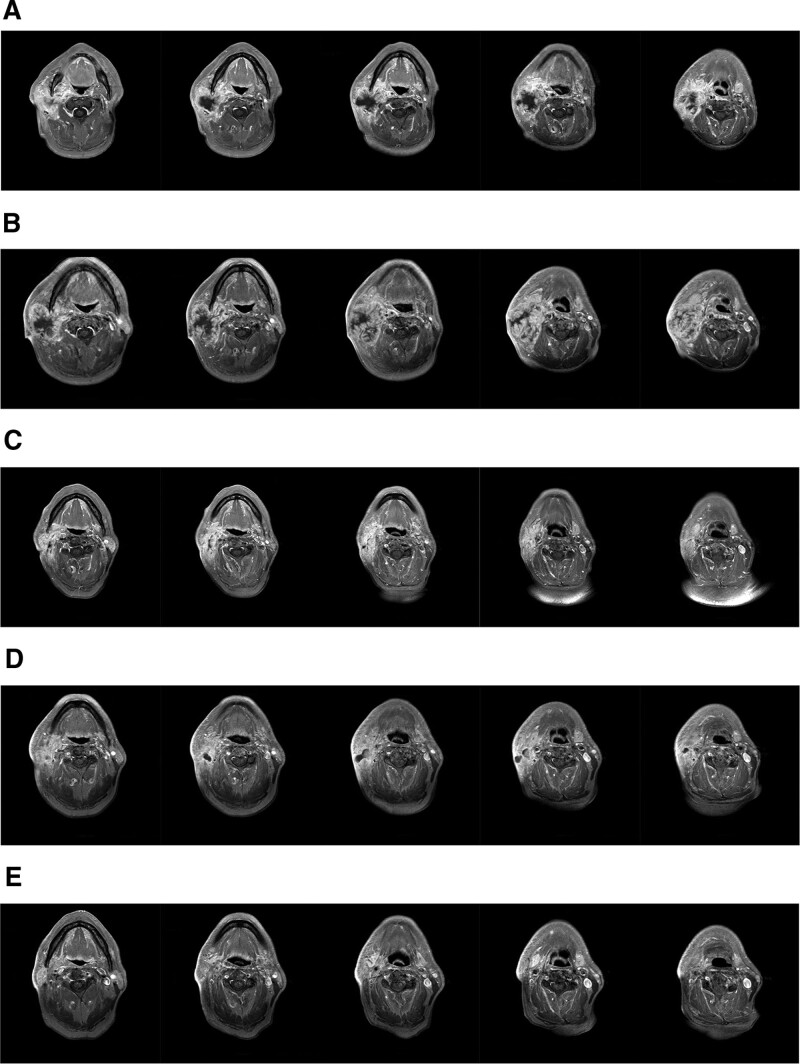
A: Head and neck MRI revealed a recurrence (January 29, 2021). B: MRI showed that the mass was larger than the previous (February 19, 2021). C: MRI revealed that the metastasis was significantly receded (March 16, 2021). D: MRI detected that the tumor has shrunk by 80% compared to the previous (April 8, 2021). E: Head and neck MRI revealed CR (May 24, 2021). CR = complete response, MRI = magnetic resonance imaging.

A 78-year-old man complaining of a lump (6*4 cm) gradually swelling on the right side of his neck with recurrent buccal cancer presented to our institution on January 28, 2021. Four months prior, a lump (2 cm in diameter) was observed on the right cheek but was not considered serious until the mass continued growing after 2 months. The neck mass was biopsied and demonstrated squamous cell carcinoma. He received resection of the buccal lesion and palatal flap repair on November 9, 2020. The operation was carried out successfully with negative surgical margins and the patient recovered well. The postoperative pathological report revealed a diagnosis of moderately differentiated keratinizing squamous cell carcinoma (Fig. 1). The patient refused suggested adjuvant chemoradiotherapy after the operation. He was a nonsmoker, did not drink alcohol and had no family history of cancer. On admission to our institution, he complained of pain, fatigue, inappetence and weight loss and was administered Tramadol (50 mg, bid) to relieve the pain. Magnetic resonance imaging (MRI) on January 29, 2021 revealed a mass in the right neck and edema of soft tissue around the right parotid gland (Fig. 2A). A neck mass biopsy revealed squamous cell carcinoma and was recognized as buccal mucosa squamous cell carcinoma cT4N1M0, stage IV according to the American Joint Committee on Cancer Staging Manual, 8th edition. RGFR amplification (copy number, 6) was detected by next-generation sequencing, with no other mutations such as copy number variations in NTRK, ALK, ROS1, and MET.

**Figure 3.**
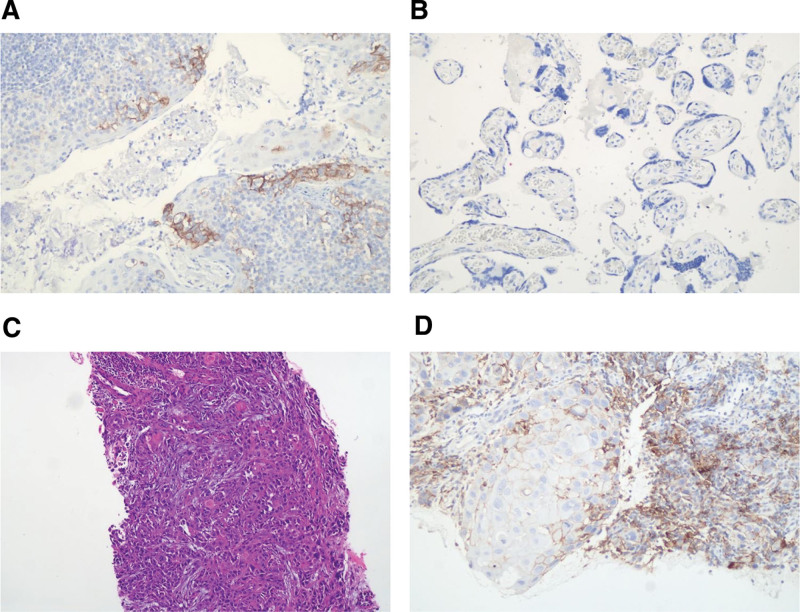
PD-L1 immunohistochemical test results: TPS = 2%, CPS = 3. A: Positive control × 200. B: Negative control × 200. C: Sample H&E × 100. D: Sample IHC × 200. CPS = combined positive score, H&E = hematoxylin-eosin staining, IHC = immunohistochemistry, TPS = tumor proportion score.

The multidisciplinary team (Head and Neck Surgery, Oncology, Radiotherapy, Pathology, and Imaging Departments) discussed that he was unsuitable for surgical treatment and determined the combined treatment scheme of immunotherapy, chemotherapy and radiotherapy. Considering his age, poor physique and positive immunohistochemical analysis of PD-L1 (TPS = 2%, CPS = 3) (Fig. 3), the patient underwent 1 cycle of induction immunotherapy with Tislelizumab (200 mg) on February 2, 2021. Two hours after the injection, the patient developed high fever (39.7°C; 103.64°F) and neck swelling. Paracetamol (500 mg, q12), caffeine (65 mg, q12), and cooling fluid infusion treatment was administered and the patient’s fever subsided the next day.

**Figure 4.**
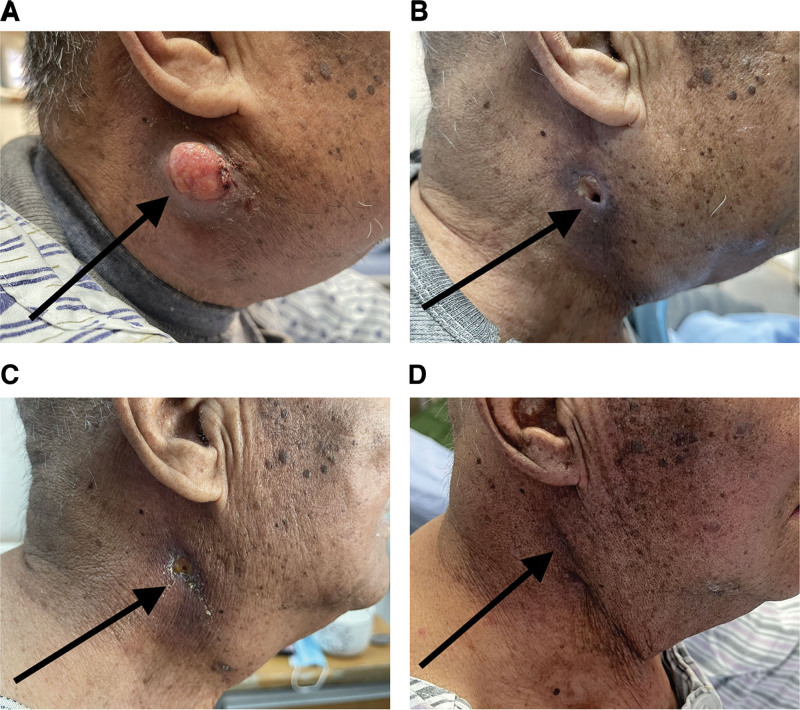
A: New organism on the surface of the neck mass. B: The new organism dissolved, forming a soft tissue sinus, with light yellow secretions flowing out of the sinus tract. C: The tumor receded and the sinus was gradually closed. D: Disappearance of the lump.

Reexamination of MRI on February 19, 2021 showed that the metastasis of the right upper and middle neck was more significant than the previously observed (Fig. 2B) and a new node appeared on the surface of the neck mass (Fig. 4A). We considered the possibility of pseudoprogression caused by the first cycle of immunotherapy and gave him another cycle of Tislelizumab (200 mg) On February 22, 2021.

On March 16, 2021, MRI revealed that the upper right neck metastasis had significantly receded compared to the front (Fig. 2C), and we found that the neck mass was significantly reduced, with the new node on the surface of the neck mass dissolving, forming a soft tissue sinus, with light yellow secretions flowing out of the sinus tract (Fig. 4B). Given that the patient tolerated the treatment better than previously, we gave him 2 cycles of Tislelizumab (200 mg) combined with Albumin-bound paclitaxel (400 mg) since March 16, 2021. The tumor receded and the sinus was gradually closed (Fig. 4C). reexamination of MRI on April 8, 2021 suggested that the tumor has considerably shrunk compared to the previous image (Fig. 2D).

Encouraged by the curative effect, we performed radiotherapy of the neck lesion from April 14, 2021 to May 28, 2021. The intensity-modulated radiation therapy plans were adopted (dose total: gross tumor volume: 70 Gy/32F/44D; clinical tumor volume: 54 Gy/30F/42D) combined with 2 cycles of Tislelizumab (200 mg) on May 6, 2021 and May 27, 2021.

**Figure 5.**
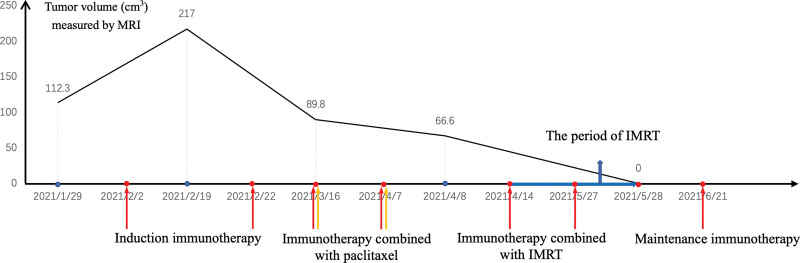
Timeline of tumor volume (cm^3^) measured by MRI and antitumor regimens. Immunotherapy (red), chemotherapy (yellow) and radiotherapy (blue). IMRT = intensity-modulated radiation therapy, MRI = magnetic resonance imaging.

The patient experienced an excellent recovery with the disappearance of pain and the lump (Fig. 4D), along with the return of a healthy appetite, weight gain and positive outlook. CR was also noted by MRI scan on May 28, 2021, with the upper right neck mass significantly retreated to unclear display (Fig. 2E). On June 21, 2021, he received 1 cycle of maintenance treatment with Tislelizumab (200 mg) which was well tolerated. The patient is still alive with stable, asymptomatic disease at the time of this writing (Fig. 5).

## 3. Discussion

We found an extremely rare case of visible pseudoprogression in HNSCC with the treatment of immunotherapy. In this case, the HNSCC patient with not obviously strong PD-L1 expression (TPS = 2%, CPS = 3) undergoing chemo-radio-immunotherapy achieved CR both clinically and radiologically.

A CR was observed in this case with immunotherapy throughout the whole process of therapy. The study of Semrau et al^[5]^ demonstrated that double immune checkpoint inhibitor (ICI) increased the response rate to induction chemotherapy for HNSCC. Wu et al^[6]^ exhibited a case confirming the safety and effect of combining anti-PD-1 antibody and chemotherapy for senile patients with recurrent HNSCC. We made a new attempt by applying the combination of Tislelizumab and paclitaxel as induction therapy and achieved sound results. The application of ICI strengthens the anti-tumor effect of radiotherapy,^[7]^ which has been supported by many reports.^[8,9]^ In this case, the patient responded well to radiotherapy combined with Tislelizumab and underwent maintenance immunotherapy to prevent tumor recurrence.

Pseudoprogression is the radiologic appearance of an increase in the size of tumor or tumor burden after ICI with subsequent tumor reduction.^[4]^ The incidence of pseudoprogression is roughly 10%,^[10]^ which was initially noted in anti-CTLA-4 therapy for melanoma^[11]^ and then reported in non-small cell lung cancer, urothelial cancer and renal cancer.^[12]^ In HNSCCs, pseudoprogression has also been reported, although it is rare,^[4]^ with an incidence of about 1.3%.^[13]^ The potential mechanism of pseudoprogression is that immune cells flow into the tumor micro-environment due to the reactivation of the immune system.^[13]^ Therefore, extensive hemorrhage and inflammatory exudate in the tumor tissue lead to necrosis or/and cell death, eventually forming the appearance of significantly enlarged lesions.^[14]^ The phenomenon of pseudoprogression has prognostic implications by benefits patients with a reduction in tumor burden.^[10]^

According to the time at which the tumor shrinks, pseudoprogression is categorized as early and delayed pseudoprogression; the former is defined as a ≥ 25% increase in tumor burden at imaging assessment within 12 weeks from the start of immunotherapy but is not confirmed as progressive disease at the next imaging assessment, whereas the latter is defined as a ≥ 25% increase in tumor burden at any imaging assessment after 12 weeks but is not confirmed as progressive disease at the next imaging assessment.^[15]^ In this case, the lump expanded after the first cycle of induction immunotherapy with Tislelizumab. Since the patient was generally in good clinical condition, we considered the presence of pseudoprogression and continued with immunotherapy. Over time, radiographic follow-ups confirmed our opinions. According to iRECIST criteria, physicians are encouraged to adhere to immunotherapy with a close imaging follow-up (no less than 4 weeks later and no longer than 8 weeks later) for patients with a generally good clinical condition or a better Karnofsky performance status score whose clinical status has not deteriorated.

Several questions are raised: first, a more reliable method is urgently needed for the diagnosis of pseudoprogression. Second, what is the appropriate duration of induction and maintenance immunotherapy? Finally, is it feasible to reduce the radiation dose of radiotherapy when concurrent with immunotherapy? Further investigation is needed to explore the potential of immunotherapy.

## 4. Conclusion

The patient with recurrent, unresectable buccal cancer achieved CR treated with chemo-radio-immunotherapy in which visible pseudoprogression was observed. These results provide confidence in the safety and efficacy of radical chemo-radio-immunotherapy for the treatment of recurrent, unresectable or metastatic HNSCC.

## Author contributions

**Investigation:** Jiajia Gu, Jingyuan Zhang, Dayong Gu, Xiuming Zhang, Feijiang Wang, Jie Luo.

**Methodology:** Jiajia Gu, Jingyuan Zhang, Dayong Gu, Xiuming Zhang, Feijiang Wang.

**Project administration:** Jiajia Gu, Jingyuan Zhang, Dayong Gu, Xiuming Zhang, Feijiang Wang, Jie Luo.

**Writing – original draft:** Jiawei Zhou.

**Writing – review & editing:** Xia He, Lirong Wu.
